# Paediatric ophthalmology in Nepal

**DOI:** 10.1038/s41433-023-02632-7

**Published:** 2023-07-03

**Authors:** Parikshit Gogate, Saibaba Saravanan, Rishi Raj Borah, Reeta Gurung, Sailesh Kumar Mishra, Yuddha Dhoj Sapkota, Srijana Adhikari, Kabindra Bachracharya, Purushottam Joshi

**Affiliations:** 1 Community Eye Care Foundation, Dr. Gogate’s Eye Clinic, Pune, India; 2 Department of Ophthalmology, D.Y.Patil Medical College, Pimpri, Pune, India; 3 School of Health Sciences, Queens University, Belfast, United Kingdom; 4 Prashasa Health Care Consultancy, Hyderabad, India; 5 Orbis International Inc, New York, United States of America; 6 Tilganga Eye Institute, Kathmandu, Nepal; 7 Nepal Netra Jyoti Sangh, Kathmandu, Nepal; 8 International Agency for the Prevention of Blindness, South East Asia, Kathmandu, Nepal; 9 Lumbini Eye Institute & Research Center, Siddharthanagar, Nepal; 10 Mechi Eye Hospital, Birtamode, Nepal

**Keywords:** Epidemiology, Education

Nepal is a landlocked country between India and China; it straddles the great Himalayas and is one of the most mountainous countries in the world. It is the fourth poorest country in Asia and its geography poses major challenge for economic development and healthcare delivery [1, 2]. Ophthalmology in Nepal came to age in 1980s after a large prevalence of blindness survey [3]. A total of 17,423 children aged 0–15 were examined in the National Blindness Survey in 1981 and the prevalence of bilateral blindness in 10–19 year olds was 0.14 per hundred. The high prevalence of blindness lead to establishment of Nepal Netra Jyoti Sangh (Organisation for service to eye & vision in Nepal, NNJS), a national NGO owing the Government responsibility, to set up secondary care hospitals in the country, with the help of international community [4]. Fourteen large eye hospitals were setup in the five zones of Nepal. The zones later gave way to seven provinces in 2016. Most of the hospitals are located in the ‘Terai’ region near the Indian border (many within walking distance!). Most of them serve patients also from India. The districts of India bordering Nepal belong to the states of Uttarakhand, Uttar Pradesh, Bihar and West Bengal and are some of the poorest parts of India with little healthcare and eye care (http://censusnepal.cbs.gov.np/Home/Index). The Nepal Netra Jyoti Sangh and Tilganga Hospital have brought about a revolution in eye care in this mountainous country next to the roof of the world. The hospitals put together performed almost three hundred and fifty thousand surgeries in 2019 (from 11,002 in 1995) for a country that has a population of less than 30 million and per capita of US$ 1071.1 in 2019 (It was US$ 129.6 in 1980) (https://data.worldbank.org/indicator/NY.GDP.PCAP.CD?locations=NP) [5]. The mountainous region has less eye care, but it also has sparser population. The valleys in middle Himalayas have the major towns of Nepal, each with its own eye centre. The Terai region at the Himalayan foothills, bordering India, have the densest population, and most eye care centres (Fig. 1).

**Fig. 1:**
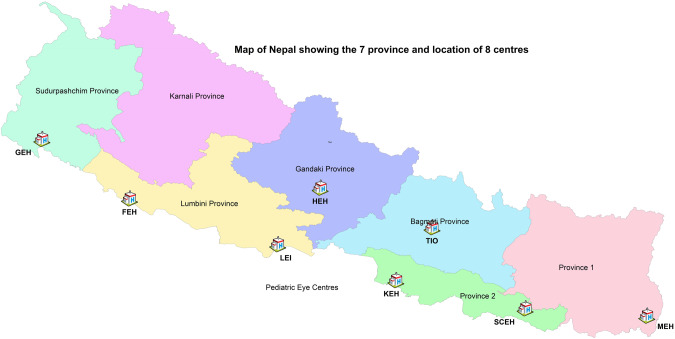
Map of Nepal with seven provinces and eight eye care centres.

Nepal pioneered the use of ‘ophthalmic assistants’ - technicians, who had done three years of training after their school leaving, to help deliver eye care. Nepal Netra Jyoti Sangh started numerous primary centres, one in each district, which would be linked to secondary hospitals. The primary eye centres were many a times embedded in primary health centres, did regular out-patient clinics, performing refraction, medicine and spectacle dispensing, as well as selection of patient requiring surgery, pre-operative workup and post-operative care for patients with cataract surgery (www.orbis.org, Accessed 5 Nov 2021). Manned by ophthalmic assistants, they form the backbone of community eye care services in Nepal.

Nepal now has twenty-three medical schools, from just one twenty years ago. The five and half year syllabus includes one year of internship. The ophthalmology residency is three years. There were four medical schools/hospitals offering post-graduate ophthalmology training, producing 35 ophthalmologists a year.

Fellowship training was getting more common, as two centres, on at Lumbini and another at Tilganga in the capital Kathmandu, are offering it. Table 1 shows the development in human resource available for eye care in Nepal.

**Table 1:** Human resource for eye care in Nepal.

Human resource	1980	2000	2010	2020	HR: Population	WHO recommended
Ophthalmologist	7	76	147	364	1: 80,046	1:50,000
Paediatric ophthalmologist	0	0	3	20		
Optometrist	0	5	36	857	1:33,999	1:50,000
Ophthalmic Assistant	0	161	275	1246	1:23384	1:25,000
Eye Health workers	0	52	90	369	1: 1850636	
Orthoptist	1	3	8	16	1: 80244	

Source: Human resource in eye care – NNJS & Government of Nepal, Vision 2020 document.

The international community has donated equipment, infrastructure and trained human resource for past thirty years. Many eye care centres have been set up. Orbis international has made yeoman contribution in the past 15 years, helping nurture paediatric ophthalmology departments linked to amelioration of childhood blindness initiatives in eight institutions [6]. Others include Seva Foundation, USA, Seva Canada Society, Christoffel Blinden Mission (CBM) International, Norwegian Association of Blind and Partially Sighted, Eye Care Foundation, Lions Club International Foundation, World Health Organization, DAK Foundation, Fred Hollows Foundation, Himalayan Cataract Project, Department for International development United Kingdom, PEEK Vision, Himalayan Cataract Project, Association for Ophthalmic Cooperation in Asia (AOCA) and Nippon 24 h Television, Swiss Red Cross, International Trachoma Initiative, Vision Himalaya, Tej Kohli & Ruit Foundation, Swiss Red Cross, USAID Envision, Government of India and Government of Japan. Eye related research from Nepal has become more common and is increasingly being done by Nepalese ophthalmologists themselves. Dr Albrecht Henning from Lahan and Dr. Sanduk Ruit from Kathmandu have pioneered new techniques in cataract surgery (https://data.worldbank.org, Accessed 5 November 2021) [7, 8]. The eye care is offered at very subsidised price to the populace, and completely free to those who cannot afford it. It’s a great example of how local doctors and philanthropist teamed up with the international community to give world class eye care to one of the poorest and remote populations in the world.

The eight paediatric eye care centres have screened 1,647,530 children, performed 56,688 surgeries (from 2010–2019) and trained 7 paediatric ophthalmologists, 3 paediatric anaesthesiologists, 18 optometrist/ophthalmic assistants, 19 ophthalmic nurses, 15 paediatric counsellors, 11 ophthalmic nurses in outreach, 5 bio medical assistants and 9 administrators. There has been a significant development of the sustainable paediatric eye care service in Nepal in terms of service availability, accessibility, human resource development and skill enhancement opportunity, service output and outcome after the Orbis supported paediatric eye care program.

This revolution in children’s eye care has been a part of a larger development story. The life expectancy in Nepal in 1981 was 48 years, the estimate for the year 2021 was 69 years, a 21-year increase in past forty years [9]. The Infant Mortality Rate in 1981 was 114, the predicted rate for year 2021 was 42. This has been due to the increase in measles vaccination coverage from 2% in 1981 to estimated 86% in 2021 and Vitamin A supplementation from 0% in 1981, to an estimated 97% in 2021 [10]. A population based study in three ecological regions of Nepal found childhood blindness prevalence to be 0.07% and severe visual impairment to be 0.1% [11]. Girls, undernourished children and those with systemic co-morbidity were more likely to be blind as well as those from the Terai region. The most common cause of blindness was amblyopia (42%) followed by congenital cataract. Corneal opacity was the commonest cause of unilateral blindness [12]. It is also estimated that due to the expansion of the Expanded Programme of Immunisation, the high coverage of measles vaccination (86%) and the vitamin A supplementation scheme (90%), the incidence of nutritional blindness must have reduced considerably.

Each country’s challenges in paediatric ophthalmology are unique and so are its response to meet them [13–22]. Many Asian countries have experienced growth of paediatric ophthalmology as a sub-speciality in past two to three decades [12–15], unlike those in Europe where the process started few decades earlier [16–19]. The Nepal experience shows that it is possible to deliver quality eye care to children in one of the poorest parts of the world, with cooperation between medical fraternity, government, non-governmental organisations, international developmental organisations and community participation and support. Its lessons may be followed in poorer regions elsewhere.

## Summary

### What was known before

- Nepal is a poor mountainous country whose level of development and geography poses a challenge for paediatric eye care delivery.

### What this study adds

- Concerted efforts of non-governmental organisations and international community has resulted in significant improvement of human resource and facilities for eye care delivery in Nepal leading to reduction in childhood blindness.
